# *Staphylococcus aureus* surgical site infection rates in 5 European countries

**DOI:** 10.1186/s13756-023-01309-w

**Published:** 2023-09-19

**Authors:** Sibylle C. Mellinghoff, Caroline Bruns, Markus Albertsmeier, Juliane Ankert, Louis Bernard, Sofia Budin, Camille Bataille, Annika Y. Classen, Florian B. Cornely, Elodie Couvé-Deacon, Maria Fernandez Ferrer, Jesús Fortún, Alicia Galar, Eva Grill, Thomas Guimard, Jürgen A. Hampl, Sebastian Wingen-Heimann, Juan P. Horcajada, Felix Köhler, Carolin Koll, Joan Mollar, Patricia Muñoz, Mathias W. Pletz, Jule Rutz, Jon Salmanton-García, Harald Seifert, Ferdinand Serracino-Inglott, Alex Soriano, Jannik Stemler, Janne J. Vehreschild, Tim O. Vilz, Jan-Hendrik Naendrup, Oliver A. Cornely, Blasius J. Liss

**Affiliations:** 1 Department I for Internal Medicine, Excellence Centre for Medical Mycology (ECMM), University Hospital Cologne, Kerpener Str. 62, 50937 Cologne, Germany; 2 Faculty of Medicine and University Hospital Cologne, Translational Research, Cologne Excellence Cluster On Cellular Stress Responses in Aging-Associated Diseases (CECAD), University of Cologne, Cologne, Germany; 3 German Centre for Infection Research (DZIF), Partner Site Bonn-Cologne, Cologne, Germany; 4 Department of General, Visceral and Transplantation Surgery, LMU University Hospital, Ludwig-Maximilians-Universität Munich, Munich, Germany; 5 Institute of Infectious Diseases and Infection Control, University Hospital Jena, Jena, Germany; 6 Service de Médecine Interne et Maladies Infectieuses, Centre Hospitalier Régional Universitaire de Tours, Tours, France; 7 INSERM, CHU Limoges, UMR 1092, Université Limoges, Limoges, France; 8 Department of Infectious Diseases, Hospital del Mar, Institut Hospital del Mar d’Investigacions Mèdiques (IMIM), Universitat Pompeu Fabra, Barcelona, Spain; 9 Centre for Biomedical Research in Infectious Diseases Network (CIBERINFEC), Instituto de Salud Carlos III, Madrid, Spain; 10 Infectious Diseases Department, CIBERINFEC, Hospital Ramón y Cajal, Madrid, Spain; 11 Department of Clinical Microbiology and Infectious Diseases, Hospital General Universitario Gregorio Marañón, Madrid, Spain; 12 Institute for Medical Information Processing, Biometrics and Epidemiology, Ludwig-Maximilians-Universität München (LMU) Munich, Munich, Germany; 13 Service de Médecine Post-Urgence, CH Départemental de Vendée, La Roche Sur Yon, France; 14 Faculty of Medicine and University Hospital Cologne, Center of Neurosurgery, Department of General Neurosurgery, University of Cologne, Cologne, Germany; 15 FOM University of Applied Sciences, Cologne, Germany; 16 Department II of Internal Medicine and Centre for Molecular Medicine Cologne, Faculty of Medicine and University Hospital Cologne, University of Cologne, Cologne, Germany; 17 Preventive Medicine Department, La Fe University and Polytechnic Hospital, Valencia, Spain; 18 Institute for Medical Microbiology, Immunology and Hygiene, University Hospital Cologne, Cologne, Germany; 19 Manchester Academic Health Science Centre, Manchester University NHS Foundation Trust, Manchester, UK; 20 Department of Infectious Diseases, Clinic Barcelona, University of Barcelona, IDIBAPS, CIBERINF, Ciber in Infectious Diseases, Barcelona, Spain; 21 Department of Internal Medicine, Hematology and Oncology, Faculty of Medicine and University Hospital of Frankfurt, Goethe University, Frankfurt, Germany; 22 Department of Surgery, University Hospital Bonn, Bonn, Germany; 23 Faculty of Medicine and University Hospital Cologne, Clinical Trials Centre Cologne (ZKS Köln), University of Cologne, Cologne, Germany; 24 Department I of Internal Medicine, Helios University Hospital Wuppertal, Wuppertal, Germany; 25 School of Medi-Cine, Faculty of Health, Witten/Herdecke University, Witten, Germany

**Keywords:** Surgical site infection, *Staphylococcus aureus*, Hospital acquired infection

## Abstract

**Objective:**

To determine the overall and procedure-specific incidence of surgical site infections (SSI) caused by *Staphylococcus aureus* (*S. aureus*) as well as risk factors for such across all surgical disciplines in Europe.

**Methods:**

This is a retrospective cohort of patients with surgical procedures performed at 14 European centres in 2016, with a nested case–control analysis. *S. aureus* SSI were identified by a semi-automated crossmatching bacteriological and electronic health record data. Within each surgical procedure, cases and controls were matched using optimal propensity score matching.

**Results:**

A total of 764 of 178 902 patients had *S. aureus* SSI (0.4%), with 86.0% of these caused by methicillin susceptible and 14% by resistant pathogens. Mean *S. aureus* SSI incidence was similar for all surgical specialties, while varying by procedure.

**Conclusions:**

This large procedure-independent study of *S. aureus* SSI proves a low overall infection rate of 0.4% in this cohort. It provides proof of principle for a semi-automated approach to utilize big data in epidemiological studies of healthcare-associated infections.

*Trials registration* The study was registered at clinicaltrials.gov under NCT03353532 (11/2017).

**Supplementary Information:**

The online version contains supplementary material available at 10.1186/s13756-023-01309-w.

## Introduction

Surgical site infections (SSI) are still among the most frequent healthcare associated infections (HAI) and entail significant morbidity and mortality globally [1]. Considering antimicrobial resistance (AMR) a global threat, mutual international efforts must focus on reducing infection rates of key players such as SSI. In Europe, the most common causative pathogen is *Staphylococcus aureus* being part of the human skin microbiota [2]. *S. aureus* SSI is associated with prolonged duration of hospitalization, death rates and treatment costs [3]. In contrast to infections caused by antibiotic-resistant organisms like methicillin-resistant *S. aureus* (MRSA), S*. aureus* SSI rates are independent on regional epidemiological influences [1].

Recent efforts to understand and reduce SSI could diminish infection rates [2, 4–6]. In the light of individualized medicine also growing in the field of infectious diseases, detailed insights into epidemiology of different patient populations are urgently needed. This may enable the development of more targeted prevention approaches. Current epidemiological studies measure SSI rates in select indicator procedures assumed representative for surgical subspecialties. This approach has been questioned by data from single institutions or provider networks suggesting relevant SSI rate variability within surgical disciplines and limited applicability of risk criteria in multiple procedure types [7].

We thus established a cohort of all patients undergoing surgery at 14 high-volume surgical care centres in Europe. We included all types of surgery rather than select indicator procedures to generate a comprehensive picture of *S. aureus* SSI. Thereby, we aimed to assess the overall S. aureus SSI infection rate and, consequently, also rates within alle included procedures.

## Methods

### Study design

**Fig. 1:**
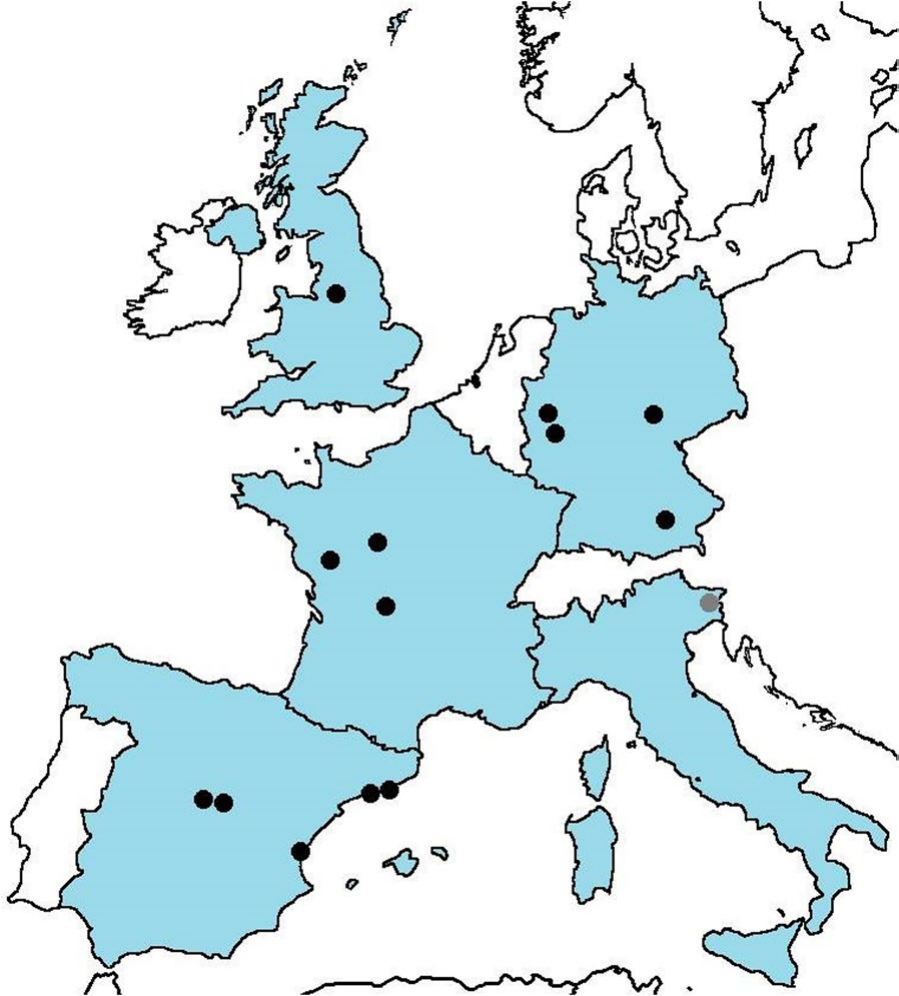
Centres participating in the SALT study. Black dots represent centres with patients in the overall cohort, as well as in the case–control analysis. Grey circle represents a centre with patients in the overall cohort. Centres by country: France (*Centre Hospitaler Universitaire de Limoges, Centre Hospitalier Régional Universitaire de Tours* and *Centre Hospitalier Départemental Vendée*), Germany (*Universitätsklinikum* Bonn, *Universitätsklinikum* Cologne, *Universitätsklinikum* Jena and *Universitätsklinikum Ludwig-Maximilians-Universität* [LMU]-Munich), Italy (*Azienda Sanitaria Universitaria* Udine), United Kingdom (Central Manchester NHS Foundation), Spain (*Hospital del Mar and Institut Hospital del Mar d’Investigacions Mèdiques* [IMIM] de Barcelona, *Hospital Clínic de Barcelona*, *Hospital General Universitario Gregorio Marañón* Madrid, *Hospital Universitario Ramón y Cajal* Madrid and *Hospital Universitari I Politècnic La Fe* Valencia). Illustration made with Biorender (C)

This is a retrospective multinational, multicentre cohort study with a nested case–control analysis. The study includes all surgical procedures performed in adult patients in 2016, excluding minimal invasive biopsies and eye surgery at 14 surgical centres in Europe (Fig. 1; Additional file 3: Table S1o ensure appropriate representation of each type of surgery, only centres with more than 10 000 annual procedures were considered. Sites were identified by their publication activity on SSI, prior SSI study participation and membership in respective European surgical, microbiological, or infectious diseases societies. Sites were contacted and selected using a feasibility questionnaire (Additional file 1).

The study was submitted to the Research Ethics Commission of the University of Cologne (No. 17–078) for advice; the requirement for informed consent was waived due to the retrospective nature as well as the anonymous data capture strategy of this study. The study was registered at clinicaltrials.gov under NCT03353532.

### Patient selection criteria

Inclusion criteria consisted of surgery in 2016 and age ≥ 18 years at the time of surgery. Exclusion criteria comprised minimal invasive biopsies and eye surgery, SSI at the time of surgery as well as cases with missing data defined as “missing completely at random” (MCAR).

Surgery was defined in analogy to established epidemiological approaches [8] as any procedure taking place in an operating room and including at least one incision. Minimal invasive needle biopsies without incision, as well as all types of eye surgery were excluded.

### Data assessment

Data were assessed at two levels: The cohort and the case–control population. All anonymously documented data and cases were reviewed by infectious disease specialists.

Data for the entire cohort of all included patients were exported from electronic patient records. This included demographics, surgical procedure code, procedure duration, comorbidity by international classification of diseases (ICD), and wound class of all patients undergoing surgery if available.

### SSI identification

**Fig. 2:**
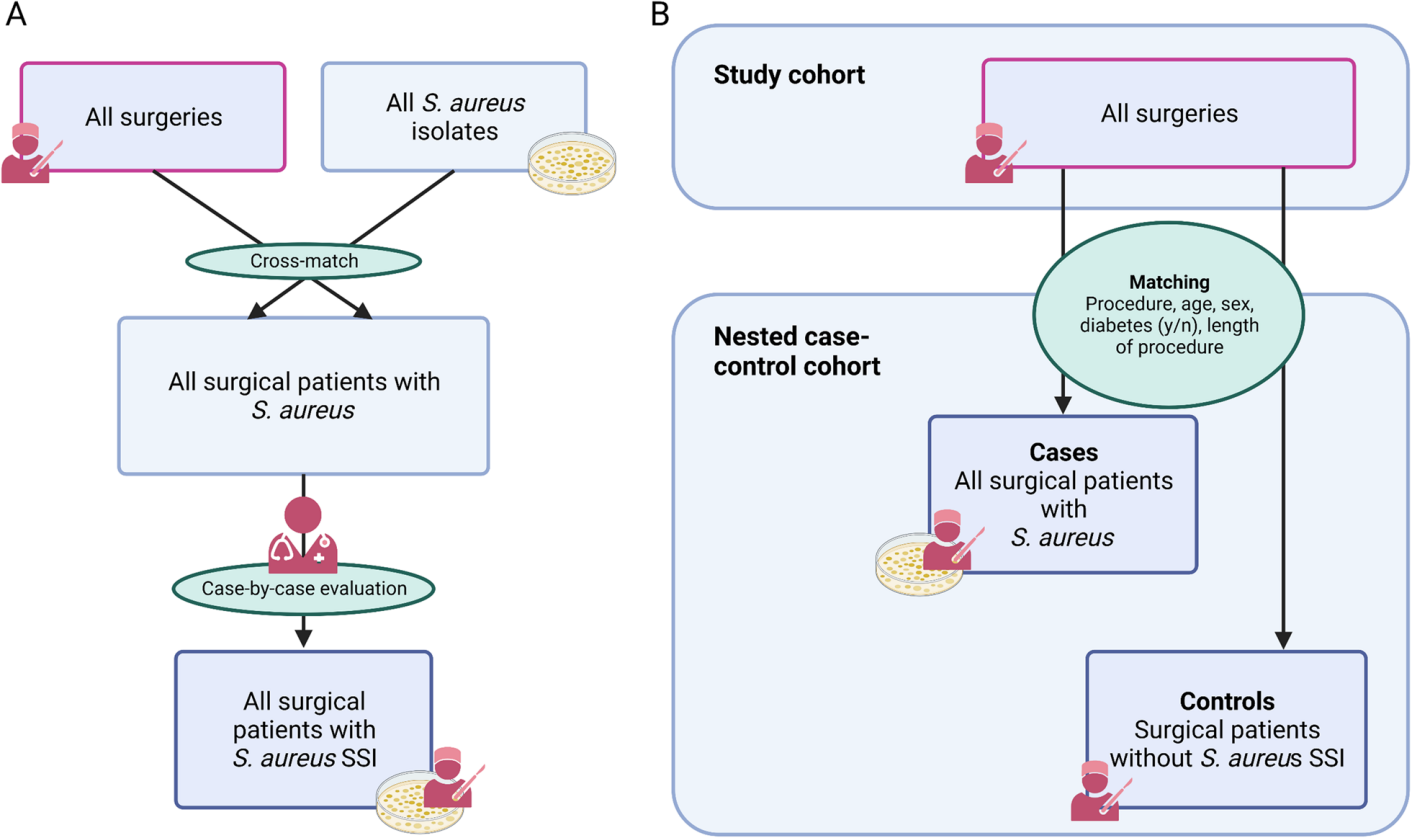
Methodology of the SALT study. A. Cases of *S. aureus* SSI were identified by crossmatching bacteriology laboratory data of all *S. aureus* isolates with data of all patients undergoing surgery, thus generating a list of all possible *S.* *aureus* SSI cases. Presence of *S. aureus* SSI among these possible cases was verified by single-case evaluation which was performed by infectious diseases specialists and surgeons in each hospital. True cases were ascertained as having *S. aureus* presence and either a documented diagnosis of SSI or as exhibiting both, a clinical picture suspicious of SSI and having undergone an intervention. Patients with non-SSI *S. aureus* (e.g., contamination, colonization, etc.) were excluded. B. To allow data assessment for outcomes, *S. aureus* SSI cases were matched to controls who underwent the same procedure using optimal propensity score matching based on cohort data, in particular age, diabetes, duration of procedure as percentile for this procedure

Cases of *S. aureus* SSI were identified by crossmatching bacteriology laboratory data of all *S. aureus* isolates with data of all patients undergoing surgery, thus generating a comprehensive list of all possible *S. aureus* SSI cases (Fig. 2). Presence of *S. aureus* SSI among these possible cases was verified by single-case evaluation which was performed by infectious diseases specialists and surgeons in each hospital to ensure inclusion of only those SA found in a relevant clinical culture. True cases were ascertained as having *S. aureus* presence and either a documented diagnosis of SSI or as exhibiting both, a clinical picture suspicious of SSI and having undergone an intervention. Patients with non-SSI *S. aureus* (e.g., contamination, colonization, etc.) were excluded.

SSI caused by pathogens other than *S. aureus* and culture-negative SSI were excluded from the study. Codes that did not comprise surgical procedures, e.g., haemodialysis, were defined by a committee of infectious disease specialists and surgeons of the respective specialties at the coordinating centre. Minimal invasive procedures and eye surgery excluded from the study are listed in the Additional file 2. Country-specific procedure codes were harmonized as described previously [9]: We included the existing surgical procedure coding systems of the five participating European countries (France, Germany, Italy, Spain, and the UK). In an iterative process, country specific codes were grouped in ever more categories until each group represented a coherent unit based on mode, interventions performed, extent and site of the surgical procedure. Next, two ID specialist (arbitrated by a third in case of disagreement) independently assigned country-specific codes to the resulting categories. Finally, specialist from each surgical discipline reviewed these assignments for their respective field. A total number of 15,432 surgical procedures were assigned to 153 codes from 10 specialties [10].

### Matching of case-controls

To allow data assessment for outcomes, *S. aureus* SSI cases were matched to controls who underwent the same procedure using optimal propensity score matching based on cohort data, in particular age, diabetes, duration of procedure as percentile for this procedure. Six of the participating centres were not equipped to export comorbidities electronically (n = 77 494). Out of all comorbidities, only the item diabetes was provided by all centres and therefore included. Before inclusion, controls were verified to be free of SSI by infectious disease experts and surgeons of the respective centres. Controls determined to have had SSI were excluded from the cohort and associated cases were rematched.

The following additional data were manually documented from *S. aureus* SSI patients (cases) and 1:1 matched patients (controls) from the same centre: American Society of Anaesthesiologists (ASA) score, body mass index (BMI), length of hospitalization, length of intensive care unit (ICU) stay, reason and attribution to SSI in case of ICU admission, survival at 30 and at 90 days, antibiotic treatment including treatment duration in days, functional status at admission and at last discharge, necessity for surgical revision, and death attributed to SSI. If readmission was necessary, reason and relatedness to SSI, length of hospitalization and ICU stay as well as all antibiotic treatments and their duration were recorded. For cases, the causative pathogens, antibiotic resistance patterns, and type of SSI according to ECDC criteria [1] were also captured.

### Statistical methods

*S. aureus* SSI incidence was defined as the number of *S. aureus* SSI per 100 surgical procedures. Based on the available literature, the *S. aureus* SSI rate was assumed to be 1.0%, independent of the surgical specialty involved and the type of procedure performed [1, 2, 4]. Thus, by observing 1,500 surgical procedures, cumulative incidences were expected to be determined with a 95% confidence interval of ± 0.5%. We therefore aimed to include 90 000 to 150 000 patients allowing to calculate incidence with a meaningful precision for all surgical procedures performed on at least 1.0% to 1.5% percent of the overall surgical population at participating centres.

The primary study objective was to determine the overall and procedure-specific incidence of *S. aureus* surgical site infections in Europe. The cohort of all patients who underwent surgery defined the denominator, while patients developing *S. aureus* SSI defined the numerator.

For the primary analysis, the cumulative incidence was calculated using 95% confidence intervals. Secondary analyses focussed on the overall and procedure-specific outcomes of *S. aureus* SSI. The dependent variable was *S. aureus* SSI. Within the nested case–control design, continuous variables are presented as mean (standard deviation) and median (interquartile range [IQR]) and compared using Student’s t-test or Wilcoxon rank-sum test after performing a normality test. We present categorical variables as proportions and compared those using Fisher’s Exact test.

We performed descriptive statistics of all parameters observed. Country-based and institution-based incidence was determined for each procedure (e.g., ventral hernia repair) and each category (e.g., vascular surgery). For each incidence, the 95% confidence intervals for a binomial proportion were calculated.

Based on case–control matching, the composition of the surgical patient population was characterized.

Further statistical analysis included comparison of SSI, in particular *S. aureus* SSI incidence, in the different participating countries. We used logistic regression to calculate odds ratios (OR).

Statistical analysis and generation of all tables, listings and figures were performed using SPSS® (IBM Corporation, Chicago, IL, USA).

### Role of the funding source

This study was an investigator-initiated trial with the University of Cologne as sponsor. The study was funded by a restricted research grant from Pfizer. The company provided advisory input into the trial design and was provided the primary raw data. Pfizer did not participate in site section, trial conduct, data analysis or manuscript writing.

## Results

**Fig. 3:**
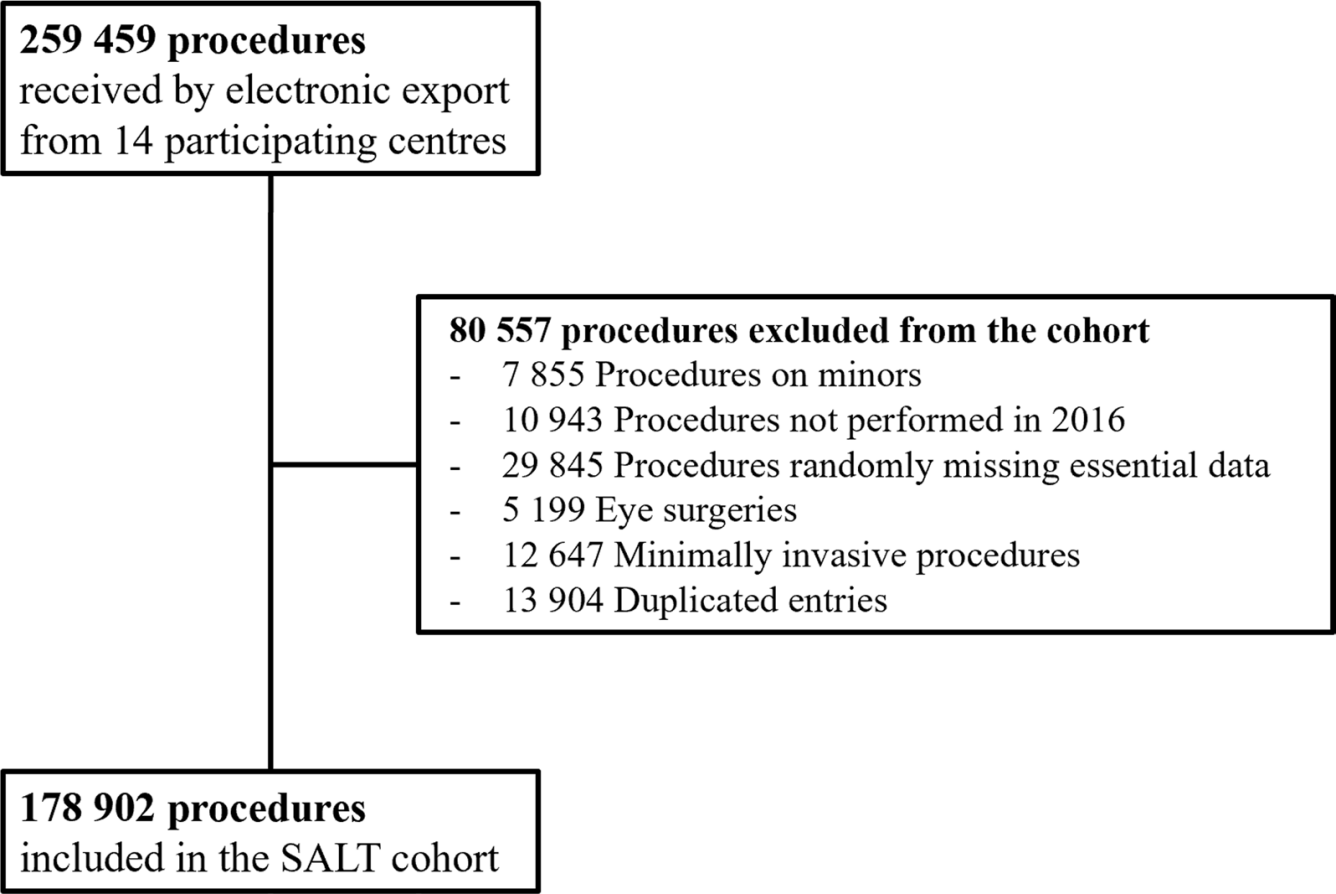
Flow chart of case inclusion

From 259 459 initially exported data sets of patients who had undergone surgery, 178 902 were included in the analysis; characteristics are depicted in Table 1. In total, 80 557 were excluded due to age < 18 years [7 855], year of surgery not 2016 (10 943), MCAR (29 845), eye or minimal invasive surgery (17 846) or duplicate entries (13 904) (Fig. 3). The procedure most frequently performed within the entire cohort was DER01 (Incision and excision of skin and subcutaneous tissue; n = 9483), followed by GYN08 (Caesarean section; n = 8146).

**Table 1:** Patient characteristics of the SALT cohort, *S. aureus* SSI cases, and controls

Characteristic	Cohort	*S. aureus* SSI cases	Controls	p-value (SSI cases vs. controls)
*Age*				
Mean	56.7	58.1 (18–95)	57.7 (18–97)	p = 0.704
*Age groups *[% (n)]				
18–29	9.5 (17,056)	9.5 (71)	11.4 (85)	
30–44	19.0 (33,967)	16.7 (124)	17.1 (127)	
45–59	23.3 (41,728)	20.4 (152)	19.6 (146)	
60–75	30.2 (53,981)	34.3 (255)	30.9 (230)	
> 75	17.98 (32,170)	19.1 (142)	21.0 (156)	
*Sex* [% (n)]				p = 0.604
Female	51.7 (92,468)	48.1 (358)	46.6 (347)	
Male	48.3 (86,434)	51.9 (386)	53.4 (397)	
*BMI* [% (n)]*	n/a			p = 0.767
< 18.5		1.9 (12)	2.2 (13)	
18.5–24.9	32.3 (204)		44.4 (263)	
25.0–29.9		34.4 (215)	32.5 (193)	
30.0–34.9		20.8 (130)	15.3 (91)	
35.0–39.9		6.9 (43)	4.0 (24)	
> 40		3.4 (21)	1.5 (9)	
*Comorbidities Cardiovascular*				
Chronic CVD	4.39 (4454/101410)	23.1 (172)	21.9 (163)	p = 0.620
Congestive HF	1.07 (1082/101410)	7.7 (57)	5.5 (41)	p = 0.117
Peripheral VD	3.43 (3482/101410)	12.1 (90)**	8.1 (60)**	p = 0.012
*Pulmonal*				
COPD	1.45 (1475/101410)	6.2 (46)	4.4 (33)	p = 0.165
*Cancer*				
Leukemia	0.15 (154/101410)	0.4 (3)	0.3 (2)	p = 1.000
Lymphoma	0.28 (285/101410)	2.2 (16)	0.9 (7)	p = 0.091
Solid tumor	7.29 (7396/101410)	22.3 (166)**	15.6 (116)**	p = 0.001
*Neurological*				
Dementia	0.32 (321/101410)	2.3 (17)	2.4 (18)	p = 1.000
TIA or CVA	0.13 (132/101410)	5.8 (43)	5.8 (43)	p = 0.591
Hemiplegia	0.89 (904/101410)	1.3 (10)**	3.1 (23)**	p = 0.033
*Other Internal*				
Diabetes	11.43 (11,591/178904)	21.0 (156)	176 (131)	p = 0.115
Liver disease	1.12 (1138/101410)	4.4 (33)	5.0 (37)	p = 0.714
CKD	3.22 (3271/101410)	7.8 (58)	7.0 (52)	p = 0.620
*Other*				
HIV/AIDS	0.09 (96/101410)	(9)	(6)	p = 0.605

^*^For BMI calculation, only 625 cases were included due to missing data in the remaining cases

^**^Statistically significant difference between *S. aureus* SSI cases and controls. For further details refer to Supp. Table 2

*AIDS* Acquired immunodeficiency disease, *CVA* Cerebral vascular accident, *CKD* Chronic kidney disease, *CVD* Chronic cardiovascular diseases, *HIV* Human immunodeficiency virus, *HF* Heart failure, *SA Staphylococcus aureus*, *SSI* Surgical site infection, *VD* Vascular diseases, *TIA* Transient ischemic attack, CVA

Data from 178 902 patients who had undergone surgery in five European countries (Fig. 1) in 2016 were analysed. Of those, 764 had *S. aureus* SSI constituting an overall incidence of 0.4% (Table 2). The overall relative proportions of superficial, deep, and organ/space SSI were 46.3%, 27.0%, and 26.7%, respectively (Additional file 3: Table S2). A total of 660 (86.0%) *S. aureus* SSIs were caused by methicillin susceptible *S. aureus* (MSSA) and 104 cases (14.0%) by MRSA.

**Table 2:** Overall incidence of *S. aureus* and of most common procedures and procedures with highest rates of SA SSI

Procedure	Number of *S. aureus* SSI (n)	Incidence of *S. aureus* SSI (%)	
All	764	0.4	
Procedure	Number of *S. aureus* SSI (n)	Procedure-specific incidence of *S. aureus* SSI (%)	Incidence by discipline (%)
Dermatological surgery Incision and excision of skin and subcutaneous tissue Wound debridement Skin autograft transplantation	87 29 28 11	0.5 0.3 0.6 1.3	3.8 3.7 1.4
Gynecological surgery Caesarean section Breast excision and resection Open surgery of ovary and fallopian tubes Open surgery on uterus and cervix uteri	121 68 15 14 5	0.5 0.8 0.4 1.3 1.3	8.9 2.0 1.8 0.7
Heart and cardiothoracic surgery Revascularization of the heart Atrial septum/valve repair surgery Open surgery of the lung and pleura (reconstruction/removal) Operation on the diaphragm	78 29 22 11 1	0.8 1.6 1.0 0.6 3.6	3.8 2.9 1.4 0.1
Neurosurgery Operations on scull, brain, meninges Insertions of neurostimulator adjacent to spinal cord	60 44 3	0.5 0.7 1.3	5.8 0.4
Orthopedic and trauma surgery Open repair of a fractured long tubular bone Other operations on bones Primary total prosthetic replacement of hip joint Total prosthetic replacement of knee joint	177 33 30 22 10	0.5 0.8 0.7 0.7 0.4	4.3 3.9 2.9 1.3
Urological surgery Minimally invasive operations on the kidney Open nephrectomy Open surgery on ureter	35 4 4 2	0.2 1.3 0.3 1.6	0.52 0.52 0.26
Vascular surgery Open embolectomy, thrombectomy and endarterectomy of blood vessels Operations on blood vessels by replacement or re-anastomosis Open insertion of stent grafts	78 14 8 9	0.6 0.7 1.0 1.6	1.8 1.1 1.2
Visceral surgery Repair of inguinal hernia Open excision and resection of pancreas Liver transplantation Minimally invasive surgery of the bile duct Laparoscopic local excision of small intestine	108 20 7 5 2 1	0.3 0.3 0.5 1.2 4.6 4.2	2.6 0.9 0.7 0.3 0.1

The highest procedure-specific incidence of *S. aureus* SSI can be reported in minimally invasive surgery on the bile duct, followed by laparoscopic local excision of the small intestine. Category-specific incidence was highest in caesarean sections, followed by operations on scull, brain, meninges and open repair of a fractured long tubular bone. A detailed list of all procedure-specific incidence can be found in the Additional file 2, *SSI* Surgical site infection

**Fig. 4:**
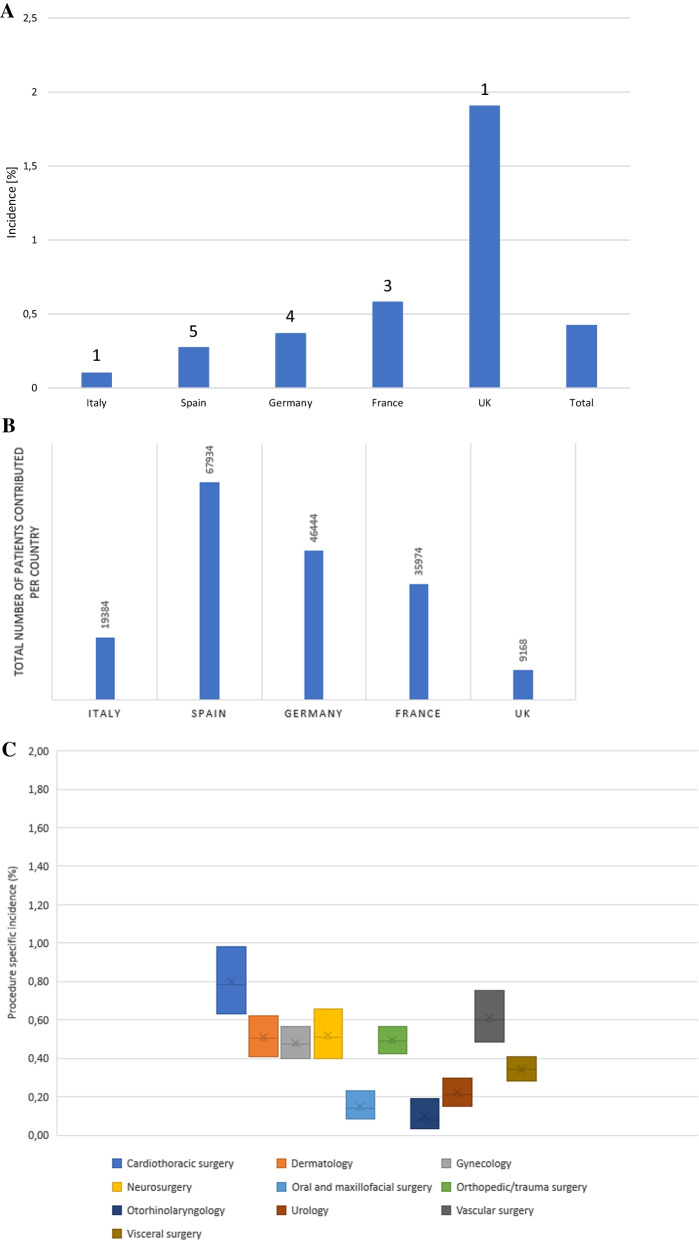
Cohort data and incidence rates of *S. aureus* SSI. **A.** Overall and country-specific *S. aureus* SSI incidence; number above columns indicate the numbers of participating centres per country. **B.** Country-specific number of patients contributed to the cohort. **C.** Incidence rates of *S. aureus* SSI by surgical discipline. Across the different surgical disciplines, *S. aureus* SSI incidence rate is 0.5 (IQR 0.3)

Cohort data and incidence of *S. aureus* SSI per country are depicted in Fig. 4. For the nested case–control part 20 (2.6%) of 764 cases were excluded due to missing documentation of the matched cases.

### Procedure-specific S. aureus SSI distribution

*S. aureus* SSI incidence did not differ across surgical specialties. Median discipline-specific incidence was 0.5 (IQR 0.265) as depicted in Fig. 4. Highest procedure-specific incidence (Table 2) was seen in visceral surgery (minimally invasive surgery of the bile duct, 4.6% [95% CI 1.2 -17.6%], and laparoscopic excision of the small intestine, 4.2% [95% CI 0.6–28.4%]) as well as cardiothoracic surgery (operation on the diaphragm, 3.6% [95% CI 0.5–24.5%]). With regard to distribution of *S. aureus* SSI, it most frequently occurred in caesarean Sects. (8.9%), operations on scull, brain, and meninges (5.8%), and open repair of fractured long tubular bones (4.32%). Surgery performed in cardiothoracic surgery (0.8%) and vascular surgery (0.6%) had a higher specialty-specific *S. aureus* SSI incidence than anticipated for the respective group along a prediction model (adjusted residual values 5.7 and 3.2, respectively). Further in depth-analyses revealed that patients undergoing surgery in one of those mentioned groups had significantly more comorbidities than patients from other surgical fields (data not shown) and were the oldest of the entire cohort (mean age in the cardiothoracic surgery group 64.3 years and in the vascular surgery group 63.3 years). A list of all procedure specific incidences is included in the Additional file 2.

### Structural equality of cases and controls

Between cases and controls, no statistically significant differences in sex, age, or BMI were observed hence indicating successful matching; distribution of comorbidities was similar and significant differences were only seen in patients with peripheral vascular diseases, hemiplegia, and solid tumours (Table 1). Cases and controls were matched along the SALT (Staphylococcus aureus Surgical Site Infection Multinational Epidemiology in Europe [SALT] study) code [10] and 760 of 764 controls had undergone the same type of procedure as the respective case. For optimal matching, four patients were allocated to similar types of procedures – three within the respective specialty (two neurosurgical procedures) and one with a surgical procedure from another specialty (dermatological procedure with one visceral surgical procedure, Additional file 3: Table S3).

Sex did not differ across the entire cohort (51.7% female and 48.3% male), but there were more men than women among SSI cases (51.9% and 48.1%, respectively; p = 0.043; Table 1). Mean age was 56.7 years for controls and 58.1 years for cases. Distribution of age groups was comparable, and in both populations the majority of patients was older than 60 years. Within the group of SSI cases, most patients (65.4%) were overweight (BMI ≥ 25). However, there was no statistically significant difference between cases and controls (p = 0.77).

The most frequent comorbidity in the overall cohort was diabetes (11.4%), followed by solid malignancies (7.3%), chronic cardiovascular disease (CVD, 4.4%), peripheral vascular disease (3.4%), and chronic kidney disease (CKD, 3.2%) (Table 1). Within the population of *S. aureus* SSI cases, CVD (23.1%), solid malignancies (22.3%), diabetes (21.0%), and CKD (7.8%) were also the most frequent comorbidities. Comorbidities of the control group were distributed in a comparable manner (significantly differing prevalence only reported for hemiplegia, solid tumours, and peripheral vascular disease; Table 1).

### Comparison of cases and controls

Univariate analyses showed that in comparison with matched uninfected controls, patients with *S. aureus* SSI were more likely to be re-admitted to the hospital (p < 0.005), more likely in need of revision surgery (p < 0.005) and hospitalized for a longer period (mean hospitalization 17 versus 12 days; p < 0.005) (Table 2). ICU stay after a surgical procedure was as frequent in cases as in controls (144 versus 130, p = 0.349). Out of 144 ICU stays in the case cohort, 42 (29.2%) were associated to SSI. An in depth-analysis of the different surgical categories showed, that case patients undergoing cardiothoracic, gynaecological, neuro- or vascular surgery were associated with significantly higher rates of readmission to hospital, revision surgery, and longer hospitalization periods (Table 3 and 4).

**Table 3:** Factors affecting likelihood of SA SSI (Logistic regression)

Factor	Pearson Chi-Square	Odds ratio (95% confidence interval)
*Cardiovascular*		
Chronic CVD	< 0.001	1.9 (1.3–2.6)
Congestive HF	0.730	1.8 (0.9–3.5)
Peripheral VD	< 0.001	2.7 (2.0–3.7)
Pulmonal		
COPD	0.108	1.6 (0.9–23.0)
*Cancer*		
Leukaemia	0.397	0.995 (0.995–0.996)
Lymphoma	0.001	3.9 (1.6–9.4)
Solid tumour	< 0.001	2.1 (1.6–2.8)
*Neurological*		
Dementia	0.673	1.4 (0.3–5.4)
TIA or CVA	0.619	1.6 (0.2–11.8)
Hemiplegia	0.061	1.9 (1.0–3.9)
*Other Internal*		
Diabetes	< 0.001	1.7 (1.4–2.1)
Liver diseases	0.102	1.7 (0.9–3.4)
CKD	0.010	1.7 (1.3–2.5)
HIV/AIDS	0.404	2.3 (0.3–16.3)

*AIDS* Acquired immunodeficiency disease, *CVA* Cerebral vascular accident, *CKD* Chronic kidney disease, *CVD* Chronic cardiovascular diseases, *HIV* Human immunodeficiency virus, *HF* Heart failure, *SA Staphylococcus aureus*, *SSI* Surgical site infection, *VD* Vascular diseases, *TIA* Transient ischemic attack

**Table 4:** Outcome of SA SSI. Complications compared between cases and matched controls without infection

Complication	Cases	Controls	p-value
Mean hospitalization (days)	17.46	11.74	p < 0.005
ICU stay following surgery	144	130	p = 0.349
Readmission to hospital	377	193	p < 0.005
Revision surgery	366	128	p < 0.005

In a multivariate analysis risk factors (Table 3) for *S. aureus* SSI were male sex, chronic cardiovascular disease (OR 1.9, confidence interval [CI] 1.3–2.6), peripheral vascular disease (OR 2.7, CI 2.0–3.7), lymphoma [OR 3.9, CI 1.6–9.4], solid tumour [OR 2.1, CI 1.6–2.8], diabetes (OR 1.7, CI 1.4–2.1) and chronic kidney disease (OR 1.7, CI 1.3–2.5). Smoking status was similar in both, cases, and controls (p = 0.774) (Table 5 and 6).

**Table 5:** Category-specific complications

Surgical category	Complication (*S. aureus* SSI cases vs controls [p-value])			
	Hospitalization (mean no of days)	ICU stay	Readmission to hospital (no of patients)	Revision surgery (no of patients)
Visceral surgery	22.6 vs 18.0 (ns)	ns	50 vs 42 (ns)	39 vs 18 (0.014)
Orthopaedic and trauma surgery	21.2 vs 14.1 (ns)	ns	107 vs 39 (< 0.005)	113 vs 33 (< 0.005)
Vascular surgery	19.2 vs 12.1 (0.018)	ns	47 vs 17 (< 0.005)	43 vs 18 (< 0.005)
Cardiothoracic surgery	25.5 vs 16.7 (0.002)	ns	28 vs 9 (< 0.005)	31 vs 7 (< 0.005)
Neurosurgery	22.9 vs 15.6 (0.004)	ns	49 vs 22 (< 0.005)	61 vs 13 (< 0.005)
Gynaecological surgery	7.6 vs 5.9 (0.065)	ns	23 vs 11 (0.015)	22 vs 3 (< 0.005)
Urological surgery	10.8 vs 5.8 (ns)	ns	14 vs 11 (ns)	7 vs 8 (ns)
Ear Nose Throat Surgery	10.8 vs 5.9 (0.062)	ns	8 vs 3 (ns)	7 vs 3 (ns)

*ns* not significant, *SA Staphylococcus aureus*, *SSI* Surgical site infection,

**Table 6:** Survival

	Cases (%)	Controls (%)	p-value
30-day survival	97.3	96.9	ns
90-day survival	93.5	95.8	p = 0.049

## Discussion

We report results from the first multinational study of surgical site infection sufficiently powered to determine SSI rates irrespective of procedure type. Our approach allowed to detect and analyse 764 cases of culture-proven *S. aureus* surgical site infections among 178 902 included patients – an investigation of a scale comparable to the most recent ECDC SSI report (1 016 *S. aureus* SSI cases) – while providing data of much higher granularity than in single-country surveillance efforts [11].

The percentage of comorbidities in our cohort mirrors the distribution among the European population, in particular the diabetes rate of 11.4% compared to 9.5% in the general population [12]. Our study population was older than the European average (37.1% above 65 years in our cohort vs 18.4% to 22.8% in the respective countries [13]) reflecting that older patients are more likely to undergo surgery [14, 15].

The overall *S. aureus* SSI incidence was 0.43%, which is about 50% lower than assumed in our sample size calculation based on European averages. While lower than the European average, our findings are in line with prior publications by centres with similar expertise (ranging between 0.2% and 0.9%) [16], [17–20] Except for a higher incidence in the UK (1.9%), we saw a uniform *S. aureus* SSI distribution among all countries – most likely reflecting similar standards among leading surgical centres. The higher incidence in the UK might be a direct result of the lower-case volume at the sole participating UK centre and a resulting true higher SSI rate. Alternatively, it might be a statistical effect as a function of the lower number of contributed cases (9 168 vs mean of 42 424). As our trial was restricted to culture proven S. aureus SSI differences in incidence might also be the result in differences in clinical approaches to SSI (e.g. the frequency of obtaining cultures or use of antibiotic prophylaxis).

In line with our hypothesis that indicator procedures are not representative of their respective categories, *S. aureus* SSI incidence displayed a high degree of intra-disciplinary variability. Currently monitored indicator procedures were representative of their respective category in some disciplines (e.g., total prosthetic replacement of knee joint 0.4% [0.2–0.7%] for orthopaedic surgery 0.5% [0.4–0.6%]), but not others (e.g., revascularization of the heart 1.6% [1.1–2.2%] for cardiothoracic surgery 0.8 [0.6–1.0%]). While statistical interference testing for each procedure was beyond the scope of this work, non-overlapping confidence intervals strongly suggest real differences rather than random effects. These differences highlight the need to expand surveillance efforts beyond indicator procedures.

Beyond our expectation of relevant *S. aureus* SSI rates in all surgical domains our data show similar average *S. aureus* SSI rates across all subspecialties. This finding is explained by *S. aureus* pertaining to the skin microbiome common to all surgical sites in contrast to SSI caused by site-specific organisms (e.g., *Enterobacterales* in GI surgery). These characteristics further strengthen our assumption that *S. aureus* is a prototypical causative organism in SSI. This supports the notion that *S. aureus* can be used as a marker or sentinel pathogen in trials focusing on overall periprocedural care and host defence in a discipline-independent fashion, i.e., in contrast to current discipline-specific approaches [1]. This finding also highlights a need for further exploration of risk factors across all procedures and subspecialties.

The identified risk factors for *S. aureus* SSI were male sex, chronic cardiovascular disease, peripheral vascular disease, lymphoma, solid tumour, diabetes and chronic kidney disease reflecting a population with severe intern diseases. As recommended in general for these patients, high awareness for infections including adequate prophylactic treatment should guide medical decisions.

The choice of our retrospective study design can be regarded as either a strength or a weakness. We believe that, in the context of HAI by a known pathogen, a retrospective study prevents the Hawthorne effect of confounding by observation while not affecting detection rates [21, 22]. This study design cannot be easily applied to the study of HAI without a microbiologically proven pathogen. Furthermore, while the choice of *S. aureus* as a marker organism is indeed compelling, our study results cannot be extrapolated to culture-negative SSI or SSI caused by other organisms. However, the current work may be regarded as a proof of concept for a novel epidemiological approach to HAI.

We limited our investigation to adult patients; our findings are thus not applicable to children. No available risk stratification approach has been established in a procedure-independent fashion and research shows wide SSI rate variability within risk categories depending on the specific surgery type [7]. Consequently, we matched cases and controls by a propensity score rather than by more conventional approaches like the NHSN SSI risk index. The paucity of variables used in matching cases and controls resulted directly from limitations of available electronic health record data and hospital information technology capabilities. However, our subsequent analysis demonstrated successful matching.

As reported elsewhere [23] our study was initially designed to establish *S. aureus* SSI patterns representative of the overall European surgical population. However, during the centre selection process it became apparent that centres with the necessary technical expertise to generate exports and perform local matching would constitute a highly select sample and thus not be representative of the overall European *S. aureus* SSI epidemiology. We thus decided to focus on analysing high performance centres rather than trying to extrapolate results from a highly select sample to the overall surgical population. We believe this approach generates more valid and robust data. *Post-hoc* changes to study design risk can introduce bias and thus compromise validity of results. This risk should, however, not apply to our analysis as we merely abstained from over-interpreting our data by trying to extrapolate it to the overall European surgical population, while not deviating in any other aspect from the trial protocol or the statistical analysis plan.

Future endeavours in applying real-world data to epidemiological research in HAI will be aided by progressive digitalization of medicine, and the compatibility of database interfaces. Technical aspects regarding the harmonization of different procedure coding systems have been discussed elsewhere [9].

## Conclusion

Our methodology and results diverge in important aspects from ongoing SSI surveillance and highlight the expanded possibilities provided by electronic health records and big data. Discrepancies between results from prospective trials and real-world evidence are a well-known issue. Contrary to prior hierarchical views of evidence levels, current approaches integrate real-world evidence with the results of more formal study types [24, 25]. We believe that such an integrative approach of supplementing prospective surveillance results with real-life data will provide a comprehensive grasp of the current state of HAI.

### Supplementary Information

**Additional file 1.** Feasibility Questionnaire for Site Selection.

**Additional file 2.** A list of all procedure specific incidences.

**Additional file 3.** Supplementary tables.

## Data Availability

All data may be available upon request to the corresponding author.
