# Supplementary material for: Staphylococcus aureus surgical site infection rates in 5 European countries
Source: Antimicrob Resist Infect Control. 2023 Sep 19;12:104. doi: 10.1186/s13756-023-01309-w (PMC10507841; doi:10.1186/s13756-023-01309-w)
Supplement: Supplementary file 1 — Additional file 1. Feasibility Questionnaire for Site Selection. [file 13756_2023_1309_MOESM1_ESM.pdf]

Welcome to SALT Feasibility Questionnaire!

Staphyloccoccus Aureus Surgical Site infection muLTinational epidemiology

For determination of the incidence of *Staphylococcus aureus* in surgical site infections (SSI) we designed a multinational, multicenter study. Our aim is to build an academic consortium to realize this project.
In addition to identification of the incidence of *Staphylococcus aureus* SSI, this study should analyze the overall and procedure specific outcome as well as the economic burden of Staphylococcus aureus surgical site infections. We will characterize the composition of the surgical patient population in Europe and estimate the number of patients at risk for Staphylococcus aureus surgical site infections. Furthermore, the economic burden in Europe, including direct and indirect costs, will be estimated.

Statistical analysis and final report will result in one or several scientific articles, mutually published in international journals.

1) How would you classify your institution?
   a. Loccal clinic
   b. Major (non-academical) surgical center
   c. Academical surgical center
   d. Speciality surgical center
2) Does your center keep electronic health records?
      i. Yes
      ii. No
3) Is your center capable of exporting an electronic list of a minimum…
   a. All surgical inpatients – including ICD codes, age, sex
      i. Yes
      ii. No
   b. All inpatient procedures – including ICPM codes and duration of procedure
      i. Yes
      ii. No
4) Is microbiological data available as electronic data?
      i. Yes
      ii. No
5) Is your center capable of exporting a list of all microbiological results?
      i. Yes
      ii. No
6) How many surgeries did your hospital perform in 2015 – or the last year with available data?
   a. < 6.000
   b. 6.000 - 10.000
   c. > 10.000

7) What kind of surgeries are performed at your hospital?

General surgery
Trauma surgery
Orthopedic surgery
Vascular surgery
Cardiothoracic surgery
Neurosurgery
Gynecological surgery
Urological surgery
ENT (Ear Nose and Throat) surgery
Plastic surgery

8) How often does your ethics committee meet?

____

9) Have you served as Principle Investigator for any clinical study before?
   i. Yes
   ii. No

10) Have you served as a Sub-Investigator before?

Thank you for participating in SALT-Feasibility Questionnaire!
