# Supplementary material for: Staphylococcus aureus surgical site infection rates in 5 European countries
Source: Antimicrob Resist Infect Control. 2023 Sep 19;12:104. doi: 10.1186/s13756-023-01309-w (PMC10507841; doi:10.1186/s13756-023-01309-w)
Supplement: Supplementary file 3 — Additional file 3. Supplementary tables. [file 13756_2023_1309_MOESM3_ESM.docx]

**Supplemental Table 1** Study Site data per country

| Characteristic | France | Germany | Italy | Spain | UK | Total |
| --- | --- | --- | --- | --- | --- | --- |
| Number of centres | 3 | 4 | 1 | 5 | 1 | 14 |
| Number of included patients | 35 974 | 46 444 | 19 384 | 67 934 | 9 168 | 178 902 |

**Supplemental Table 2** Microbiological data and wound class details of *S. aureus* SSI cases

| **Wound class** | n | % |
| --- | --- | --- |
| Superficial incisional | 354 | 46.3 |
| Deep incisional | 206 | 27.0 |
| Organ space | 204 | 26.7 |
| **Microbiological data** |  |  |
| MRSA | 104 | 14.0 |
| MRSA unknown | 7 | 0.9 |
| PVL-*S. aureus* | 1 | 0.1 |
| PVL-*S. aureus* unknown | 368 | 49.0 |
| **Clinical specimen** |  |  |
| Wound aspirate | 71 | 9.5 |
| Blood culture | 35 | 4.7 |
| Sample obtained during surgery | 200 | 26.9 |
| Wound swab | 438 | 58.9 |

Abbreviations: MRSA, methicillin-resistant *Staphylococcus aureus*; PVL-SA, Panton-Valentine leukocidin producing *Staphylococcus aureus,* SSI – Surgical site infection.

**Supplemental Table 3** Allocation of cases and controls beyond optimal matching

| Case | | | | Control | | | |
| --- | --- | --- | --- | --- | --- | --- | --- |
| Procedure (SALT Code) | Sex | Age | Name of specific  procedure | Procedure (SALT Code) | Sex | Age | Name of specific  procedure |
| NSY02 (Access to the vertebral column) | Male | 71 | Reopening of laminectomy site | NSY01 (Operations on scull, brain, meninges) | Male | 77 | Other craniotomy |
| NSY03 (Operations on spinal cord and spinal cord structures) | Male | 63 | Insertion of catheter into spinal canal | NSY05  (Insertions of neurostimulator adjacent to spinal cord) | Male | 74 | Implantation or replacement of peripheral neurostimulator lead(s) |
| DER01  (Incision and excision of skin and subcutaneous tissue) | Male | 72 | Radical and extensive excision of tissue on the skin and subcutaneous tissue with transplantation or local flap surgery | VIS08 (Open surgery on the stomach) | Male | 74 | (Total) esophagectomy with restoration of continuity |
